# Savolitinib in brain and leptomeningeal metastases from non-small cell lung cancer: a case report

**DOI:** 10.3389/fonc.2026.1627170

**Published:** 2026-04-24

**Authors:** Xuejiao Qi, Zhijiao Song, Junying He, Hui Bu

**Affiliations:** 1 Key Laboratory of Clinical Neurology, Ministry of Education, Hebei Medical University, Shijiazhuang, Hebei, China; 2 Department of Neurology, the Second Hospital of Hebei Medical University, Shijiazhuang, Hebei, China; 3 Key Neurological Laboratory of Hebei Province, Shijiazhuang, Hebei, China

**Keywords:** brain metastases, leptomeningeal metastases, mesenchymal-epithelial transition factor, non-small cell lung cancer, savolitinib

## Abstract

In the population with non-small cell lung cancer (NSCLC), the incidence of mesenchymal–epithelial transition factor exon 14 (METex14) skipping mutations is approximately 3%–4%. However, the prevalence of this mutation appears to be lower among patients with NSCLC presenting with brain metastases (BMs) or leptomeningeal metastases (LMs). Although METex14 skipping mutations confer high sensitivity to small-molecule MET tyrosine kinase inhibitor (TKI), data regarding their efficacy specifically against BM or LM remain limited. Herein, we presented the case of a 66-year-old man diagnosed with lung adenocarcinoma. METex14 skipping mutations were detected in circulating tumor DNA (ctDNA) from the cerebrospinal fluid (CSF) via next-generation sequencing (NGS). The patient derived significant clinical benefit from savolitinib treatment, achieving an intracranial partial response (PR) upon evaluation. In conclusion, NGS-based detection of ctDNA in CSF provides a valuable tool for elucidating tumor heterogeneity and the distinct molecular profiles of central nervous system (CNS) metastases compared to primary tumors. Furthermore, savolitinib demonstrated promising intracranial activity and a manageable safety profile in this METex14-altered NSCLC case. These findings suggest that savolitinib may represent a viable novel treatment option for this patient population. Collectively, our data indicate that savolitinib holds promise as a novel treatment strategy for this population.

## 1 Introduction

Central nervous system (CNS) metastases are a frequent and advanced complication of non-small cell lung cancer (NSCLC). They are estimated to occur in 20%–25% of patients with advanced NSCLC at initial presentation and develop in 30%–40% of patients during the course of their disease (1). CNS metastases include brain metastases (BMs) and leptomeningeal metastases (LMs). LM, also known as leptomeningeal carcinomatosis, involves the infiltration of metastatic cancer cells into the cerebrospinal fluid (CSF) (2, 3). Clinical trials of systemic treatments largely exclude patients with BM, especially LM. As a result, the optimal management of LM and BM is poorly defined. The prognosis of patients with NSCLC with CNS metastases is poor, and further improvement is greatly needed. The median overall survival (OS) for patients with BM is 3–7 months. However, the median OS of LM is only approximately 3 months in heavily pre-treated patients (4, 5). Male sex [hazard ratio (HR) 0.61], absence of hydrocephalus (HR 0.42), and targeted therapy post-diagnosis (HR 0.33) were independent predictors of improved survival in LM (5).

The presence of mesenchymal–epithelial transition factor exon 14 (METex14) skipping mutations in CSF is rare in patients with NSCLC with CNS metastases. Savolitinib is a highly selective oral targeted therapy for NSCLC harboring the METex14 skipping mutations. However, data on the efficacy of savolitinib in patients with CNS metastases remain limited. Here, we report a patient with NSCLC and CNS metastases harboring METex14 skipping mutations who benefited from savolitinib.

## 2 Case presentation

A 66-year-old man was admitted to our hospital in November 2022, with a 3-day history of left-sided limb weakness and slurred speech, accompanied by nausea and vomiting. A brain computed tomography (CT) scan revealed a hypodense lesion in the right frontal lobe. Although the patient’s symptoms improved slightly with antiplatelet and supportive therapy, left limb weakness and speech impairment persisted. He was initially diagnosed with cerebral infarction. He had a history of hypertension for over 10 years and pulmonary nodule resection 2 years prior. Postoperative pathology confirmed lung adenocarcinoma with a METex14 skipping mutation, for which he was on regular follow-up. He had a 20-year smoking history but had quit 2 years earlier.

**Figure 1:**
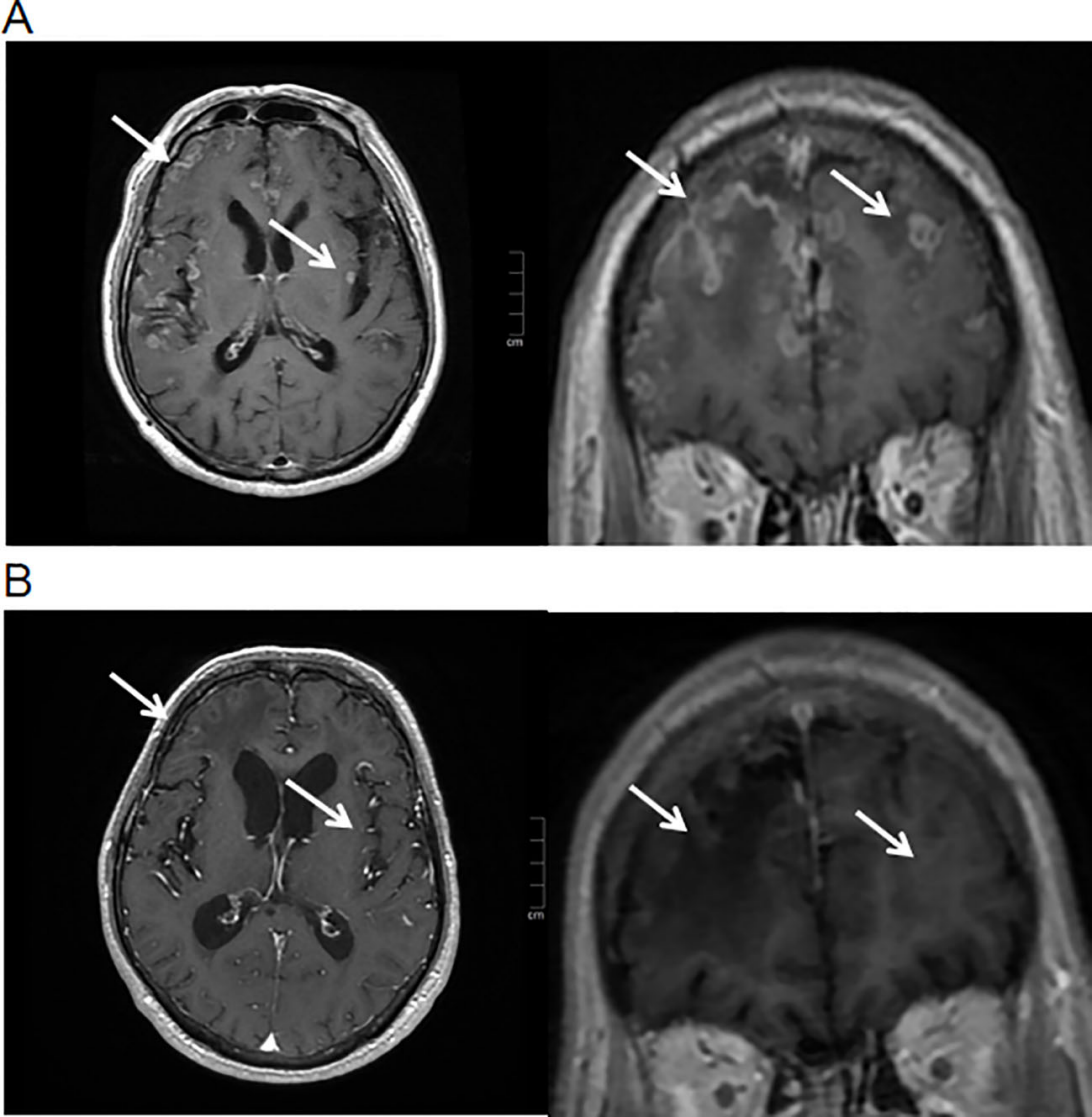
Head contrast-enhanced MRI. **(A)** This showed the nodular and ring enhancement of the fronto-temporo-parietal lobes before savolitinib treatment (white arrow). **(B)** MRI profile 8 months post-savolitinib treatment (white arrow).

**Figure 2:**
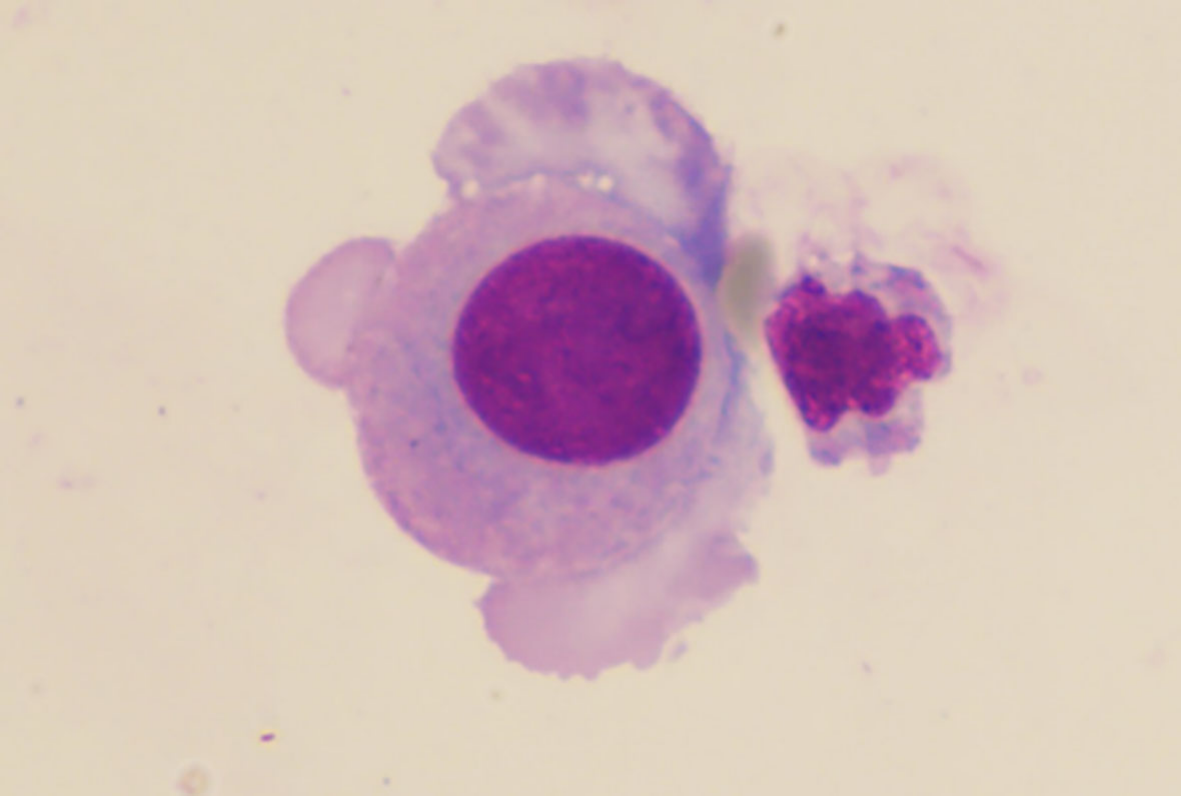
May–Grünwald–Giemsa staining of the CSF sample. The data showed the tumor cells in the CSF (×1,000).

**Figure 3:**
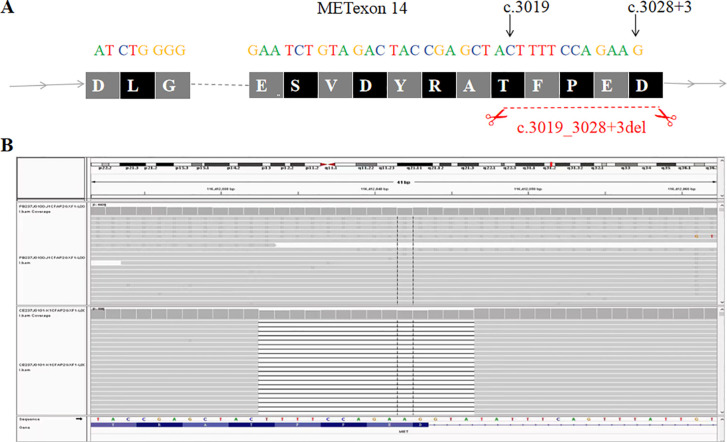
A novel MET exon 14 skipping was discovered in the CSF of a patient with NSCLC and CNS metastases. **(A)** Illustration of a METex14 variant. **(B)** Sequencing reads of MET is shown by the Integrative Genomics Viewer.

Contrast-enhanced cranial magnetic resonance imaging (MRI) showed nodular and ring-enhancing abnormalities in the bilateral frontal, temporal, and parietal lobes (**Figure 1A**). His Karnofsky Performance Status (KPS) was 50. Lumbar puncture examination of CSF revealed elevated intracranial pressure (ICP) (>330 mmH_2_O), increased white blood cell count (17 × 10^6^/L), and elevated protein level (0.46 g/L). Cytological analysis of CSF confirmed the presence of tumor cells (**Figure 2**). Next-generation sequencing (NGS) of the CSF sample rather than plasma identified METex14 skipping mutation, along with amplifications of cyclin-dependent kinase 4 (CDK4), mouse double minute 2 homolog (MDM2), and telomerase reverse transcriptase (TERT) (**Figures 3A, B**).

Based on CSF and MRI findings, the patient was definitively diagnosed with both BM and LM. Because of the patient’s refusal of radiotherapy and chemotherapy, the patient received oral savolitinib (400 mg/day), a single intrathecal injection of methotrexate (5 mg, twice a week), and supportive care. The patient experienced mild nausea and tolerable vomiting as adverse effects. Within the first 8 days of treatment, his limb weakness and speech impairment showed corresponding improvement. He was subsequently discharged from the hospital on continued oral savolitinib. The patient declined the recommended follow-up brain MRI at 2 months for metastasis monitoring, whose KPS score was 60. A follow-up brain MRI performed at 7 months after discharge demonstrated intracranial partial response (PR), and KPS score was 70 (**Figure 1B**). The patient ultimately died in October 2023 due to severe pneumonia and septic shock. His OS following the diagnosis of BM and LM was 11 months.

## 3 Discussion

This case highlights the importance of savolitinib for patients with NSCLC with CNS metastases harboring the METex14 skipping mutation. It also highlights the critical role of timely CSF NGS analysis in detecting actionable genetic alterations to guide precise treatment. Clinically, both BM and LM can mimic other neurological conditions, such as cerebral infarction, which is sometimes the initial presenting symptom in patients with underlying malignancy.

Notably, literature indicates that early-stage cerebral infarction lesions on contrast-enhanced MRI may demonstrate enhancement, suggesting the potential hemorrhage and poor prognosis (6). In contrast, BMs typically manifest on MRI as nodular or ring-enhancing lesions. These imaging features are closely associated with the histological subtype of the primary tumor and are often accompanied by extensive peritumoral edema (7). A characteristic radiological finding of BM is a “small lesion with extensive edema” (8). Given the patient’s history of lung adenocarcinoma and the presence of multiple enhancing intracranial lesions on MRI, BM was considered more likely than cerebral infarction.

The gold standard for diagnosing LM remains the cytological detection of tumor cells in CSF, with a sensitivity of 75%–90% and specificity approaching 100%. Importantly, the detection rate may exceed 90% after three repeated lumbar punctures (9). Emerging evidence also suggests that CSF-derived circulating tumor DNA (ctDNA) analysis can reveal distinct genetic profiles of intracranial metastases. CtDNA in cerebrospinal fluid aids in disease diagnosis and enhances our understanding of resistance mechanisms in patients with CNS involvement (10). Consequently, we pursued CSF ctDNA testing for further therapeutic guidance.

Interestingly, the ctDNA identified in this case revealed a METex14 skipping mutation co-amplified with CDK4 and MDM2, alterations that were detected in the CSF but not in plasma. As a key modality of liquid biopsy, ctDNA plays a pivotal role in tumor diagnosis and management (11). In patients with CNS progression, plasma-based liquid biopsies detect actionable mutations in only approximately 6% of cases, whereas CSF-derived ctDNA significantly improves detection sensitivity (12).

MET mutation is a key driver of oncogenic activation and a major mechanism of resistance to epidermal growth factor receptor tyrosine kinase inhibitors (EGFR-TKIs) in NSCLC (13). Common MET alterations include METex14 skipping mutations and MET amplification (14). In a Chinese population with NSCLC, the incidence of METex14 skipping mutations ranges from 0.9% to 2.0% (15). However, METex14 skipping mutations in CNS metastases are less reported (16, 17). In mouse models, 50% of tumors carrying the METex14 skipping mutations developed BM. In contrast, BMs were completely absent in the control group.

In this case, the patient’s primary lung adenocarcinoma harbored METex14 skipping mutations. He subsequently developed intracranial progression without evidence of active pulmonary disease. The mechanism of METex14 skipping mutations in CNS metastases is complex. Wang et al. reported that METex14 increased receptor activity and expression on cell surfaces, and prolonged downstream signaling through impairment of endocytic degradation, resulting in significantly enhanced cell migration and scattering invasion capacity, especially BM (18). Savolitinib is a highly selective MET-TKI (19). An objective response rate of approximately 50% has been observed with first-line savolitinib in patients with NSCLC harboring METex14 skipping mutations (20, 21). However, evidence regarding BM in patients with NSCLC harboring METex14 skipping mutations is primarily confined to case reports and retrospective analyses, lacking robust data from prospective trials. Follow-up brain MRI revealed a reduction in the patient after 7 months of oral savolitinib administration. Yu et al. reported 10 patients with NSCLC with METex14 skipping mutations treated with savolitinib. Nine achieved stable disease (with complete intracranial response in three) and one had extracranial progression, though the LM status was not specified (20).

Previous studies described TP53 and MDM2 as the most common co-occurring mutations in patients with NSCLC harboring METex14 skipping mutations (22). MDM2 amplification and overexpression are associated with resistance to chemotherapy and TKIs, as well as rapid disease progression following immunotherapy (23). Notably, in this case, the patient presented with amplifications of MDM2 and CDK4. This aligns with prior studies indicating that patients with METex14 skipping mutations and MDM2 amplification may not respond to crizotinib but can derive clinical benefit from savolitinib (24).

Regarding adverse effects, the patient received savolitinib 400 mg once daily and experienced only mild nausea and vomiting. Common adverse reactions of savolitinib primarily include nausea and vomiting, peripheral edema, hypoalbuminemia, hepatorenal impairment, fatigue, and hypokalemia, most of which are grade 1–2 adverse events (21). Management should be based on clinical practice and implemented through lifestyle and dietary modifications, pharmacological interventions, and treatment modification (25).

## 4 Conclusion

In conclusion, this case report describes a patient with NSCLC and CNS metastases (BM and LM) whose CSF tested positive for METex14 skipping mutations. He demonstrated clinical and radiological improvement following savolitinib treatment. This report contributes to the growing evidence on the management of patients with NSCLC with CNS metastases harboring this specific mutation. Further prospective clinical studies on savolitinib for NSCLC with CNS metastases are warranted.

## Data Availability

The original contributions presented in the study are included in the article/supplementary material. Further inquiries can be directed to the corresponding author.
